# Dietary Intake of Anthocyanidins and Renal Cancer Risk: A Prospective Study

**DOI:** 10.3390/cancers15051406

**Published:** 2023-02-23

**Authors:** Xin Xu, Yi Zhu, Shiqi Li, Dan Xia

**Affiliations:** Department of Urology, First Affiliated Hospital, School of Medicine, Zhejiang University, Hangzhou 310003, China

**Keywords:** anthocyanidins, renal cancer, PLCO, cohort, risk

## Abstract

**Simple Summary:**

In this large prospective study based on the PLCO trial, both categorical analysis and continuous analysis indicated that higher dietary anthocyanidin consumption was associated with a lower risk of renal cancer. To the best of our knowledge, this is the first prospective study that aimed to explore a potential association between dietary anthocyanidin intake and renal cancer risk.

**Abstract:**

Evidence on the association between anthocyanidin intake and renal cancer risk is limited. The aim of this study was to assess the association of anthocyanidin intake with renal cancer risk in the large prospective Prostate, Lung, Colorectal and Ovarian (PLCO) Cancer Screening Trial. The cohort for this analysis consisted of 101,156 participants. A Cox proportional hazards regression model was used to estimate the hazard ratios (HRs) and the 95% confidence intervals (CIs). A restricted cubic spline model with three knots (i.e., 10th, 50th, and 90th percentiles) was used to model a smooth curve. A total of 409 renal cancer cases were identified over a median follow-up of 12.2 years. In the categorical analysis with a fully adjusted model, a higher dietary anthocyanidin consumption was associated with a lower risk of renal cancer (HR_Q4vsQ1_: 0.68; 95% CI: 0.51–0.92; *p* for trend < 0.010). A similar pattern was obtained when anthocyanidin intake was analyzed as a continuous variable. The HR of one-SD increment in the anthocyanidin intake for renal cancer risk was 0.88 (95% CI: 0.77–1.00, *p* = 0.043). The restricted cubic spline model revealed a reduced risk of renal cancer with a higher intake of anthocyanidins and there was no statistical evidence for nonlinearity (*p* for nonlinearity = 0.207). In conclusion, in this large American population, a higher dietary anthocyanidin consumption was associated with a lower risk of renal cancer. Future cohort studies are warranted to verify our preliminary findings and to explore the underlying mechanisms in this regard.

## 1. Introduction

The incidence of and costs related to renal cancer have increased during the last two decades. As the population ages, the prevalence of established risk factors such as obesity, hypertension and chronic kidney disease increases, and the expansion of routine imaging for many disorders means that the renal cancer burden will increase significantly [1,2]. The management of renal cancer has evolved rapidly in recent years with several immunotherapy-based combinations of strategies approved as first-line therapies for the metastatic disease. However, renal cancer remains one of the most lethal urological malignancies. According to the updated data reported by the World Health Organization, there were more than 140,000 renal cancer related deaths worldwide in 2012 [3]. With rising rates of recurrence, aside from developing a personalized therapeutic treatment plan with minimal adverse events [4], it is fundamentally important to improve cancer prevention by identifying the potential factors associated with its risk.

Recent evidence has suggested that dietary flavonoid intake may be associated with decreased risk of chronic and degenerative diseases [5]. Flavonoids are classified into 12 major subclasses based on chemical structures and different subclasses may have different effects on human diseases. Anthocyanins are colored water-soluble pigments belonging to flavonoids, which provide red, blue and purple colors to fruits and vegetables. Anthocyanin pigments have been widely used as natural food colorants [6]. Recently, these colored pigments were found to have potent antioxidant properties, which give various beneficial health effects on cardiovascular [7] and neurodegenerative diseases, as reported by scientific studies from cell culture, animal models and clinical trials [8]. Similarly, dietary anthocyanidin intake has been found to be associated with a lower risk of several cancers, including lung cancer [9], head and neck cancer [10], and esophageal cancer [11]. To the best of our knowledge, evidence on the association between anthocyanidin intake and renal cancer risk is limited. An early hospital-based case-control study from Italy was undertaken on this topic and found no significant association between anthocyanidin consumption and renal cell carcinoma [12] based on 767 RCC cases and 1534 hospital controls. Therefore, the aim of this study was to assess the association of anthocyanidin intake with renal cancer risk in the large prospective Prostate, Lung, Colorectal and Ovarian (PLCO) Cancer Screening Trial.

## 2. Materials and Methods

### 2.1. Study Design and Population

The PLCO Cancer Screening Trial was a multicenter randomized controlled trial designed to assess whether screening exams could reduce the mortality from prostate, lung, colorectal, and ovarian cancers, and its study design and implementation were described previously [13]. There were two arms in the PLCO trial: the intervention arm and the control arm. A total of 76,682 men and 78,215 women aged between 55 to 74 years were enrolled in the PLCO study between November 1993 and September 2001 in ten screening centers across the United States of America (Washington, Pittsburgh, Honolulu, Denver, Marshfield, Minneapolis, Birmingham, Salt Lake City, Detroit, and St Louis). The principal recruitment strategy targeted individuals from the general population residing in the nearby areas of the screening centers. Participants in the intervention arm were screened during the first 3–4 years and participants in both arms of the study were subsequently followed for up to 10 more years to determine the potential benefits or harms of the screening exams. From its inception, the PLCO was designed not only as a RCT for the screening for four cancers but also more broadly as a research enterprise consisting of the trial, being a large, well-characterized cohort with all-cancer outcomes [14].

The current analysis is a secondary analysis of a primary database from the PLCO study. In total, 4918 participants were excluded because of a lack of baseline questionnaire data. Participants who did not complete a valid questionnaire or had been diagnosed with any cancer were also excluded (*n* = 48,237). We further excluded individuals with an implausible energy intake (i.e., lowest or highest 1%) (*n* = 546), with renal pelvis cancer (*n* = 34), and without follow-up time (*n* = 6). Overall, the cohort for this analysis consisted of 101,156 participants. Written informed consent was obtained from all the study participants, and the study protocol was approved by the Institutional Review Board of the NCI. The data used in this study were applied from the PLCO website with the permission of the NIH PLCO study group (CDAS project “PLCO-1020”).

### 2.2. Data Collection

Participants were arranged to complete a self-administered questionnaire containing personal baseline information. From the PLCO study, we collected information regarding age, gender, body mass index (BMI), race/ethnicity, education level, smoking status, and history of hypertension.

The Diet History Questionnaire (DHQ) version 1.0 (National Cancer Institute, 2007) was used to collect the dietary information, including the total daily energy intake and the daily intake of anthocyanidins. The DHQ recorded the frequency and quantity of 124 food items and supplements used over the past 12 months [15]. The daily frequency of food consumption was then multiplied by the representative sex-specific portion size of the food item using food composition data, which was based on the United States Department of Agriculture 1994–1996 Continuing Survey of Food Intakes by Individuals (CSFII) and the University of Minnesota’s Nutrition Data Systems for Research [16]. The DHQ was found to fare as well as or better than two widely used food frequency questionnaires (FFQs) when the PLCO trial was conducted [15]. Cyanidin, delphinidin, malvidin, peonidin, petunidin, and pelargonidin are the six common types of anthocyanidins [6,8]. In this study, the daily intake of these subclasses was collected through the DHQ. The amounts for processed foods were assumed to be 50% of the raw foods to account for the losses during processing [9]. The total daily intake of anthocyanidins was the sum of all six classes.

### 2.3. Renal Cancer Ascertainment

In this study, the outcome was the incidence of renal cancer. In the PLCO trial, the confirmation of the diagnosis of renal cancer was obtained from the study update forms which were mailed to participants annually asking about cancer diagnosis in the prior year. The follow-up time was based on reports from physicians and the results were confirmed by periodic linkage to the state cancer registries and the death certificates. In this study, a renal cancer case was defined as a malignant neoplasm of unspecified kidney, except for the renal pelvis (2022 ICD-10-CM Diagnosis Code C64.9). Follow-up started one year after completion of the DHQ and continued until the participants were diagnosed with cancer, withdrew from the trial, died from any cause, or completed the 10-year follow-up, whichever came first.

### 2.4. Statistical Analysis

A Cox proportional hazards regression model was used to estimate the hazard ratios (HRs) and 95% confidence intervals (CIs). The models were adjusted for potential confounders including age (continuous), sex, race (white versus non-white), BMI (<25.0 kg/m^2^ versus ≥25.0 kg/m^2^), education level (≤high school versus ≥some college), smoking status (ever versus current versus never), hypertension status (yes versus no), and total energy intake (continuous). The proportional hazards (PH) assumption was examined using the Schoenfeld residual test [17]. To assess the statistical significance of the potential differences across subgroups, Wald tests were performed on the interaction terms between anthocyanidin intake and the stratifying covariates. A restricted cubic spline model [18] with three knots (i.e., 10th, 50th, and 90th percentiles) was used to evaluate the non-linearity of the associations. In a sensitivity analysis, we excluded cases diagnosed within the first two years of follow-up and then repeated the analysis. All statistical analyses were performed using the software STATA version 15 (Stata Corp, College Station, TX, USA). All tests were two-sided.

## 3. Results

### 3.1. Study Characteristics

A total of 409 renal cancer cases were identified over a median follow-up of 12.2 years. Anthocyanidins from the diet ranged from 0 to 237.36 mg/day (median value: 12.17 mg/day). Table 1 shows the characteristics of participants by quartiles of anthocyanidin consumption. Overall, compared to participants with a lower intake of anthocyanidins, those with a higher consumption tended to be older, and were more likely to be female, non-Hispanic white, and never smokers at baseline. They also had a lower BMI but a higher rate of hypertension (all *p* < 0.001).

### 3.2. Dietary Anthocyanidin Intakes and Renal Cancer Risk

As shown in Table 2, in categorical analyses with a fully adjusted model, a higher dietary anthocyanidin consumption was associated with a lower risk of renal cancer (HR_Q4vsQ1_: 0.68; 95% CI: 0.51–0.92; *p* for trend <0.010). A similar pattern was obtained when anthocyanidin intake was analyzed as a continuous variable. The HR of one-SD increment in the anthocyanidin intake for renal cancer risk was 0.88 (95% CI: 0.77–1.00, *p* = 0.043). The proportional hazards assumption was verified using Schoenfeld residuals.

Table 3 shows the effects of the subclasses of anthocyanidin intake on renal cancer risk. The intake of delphinidin, peonidin, and petunidin was statistically significantly associated with at least a 30% reduction in the risk of renal cancer, with comparison of the highest vs. lowest quartiles (HR_Q4vsQ1_ for delphinidin: 0.59; 95% CI: 0.43–0.79; HR_Q4vsQ1_ for peonidin: 0.68; 95% CI: 0.50–0.93; HR_Q4vsQ1_ for petunidin: 0.69; 95% CI: 0.50–0.94). However, there was no significant association between the consumption of cyanidin, malvidin or pelargonidin and renal cancer risk.

### 3.3. Additional Analyses

**Figure 1:**
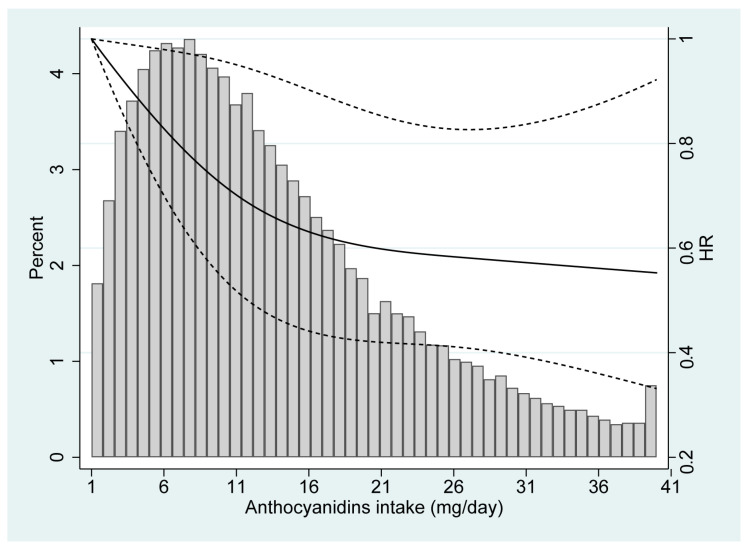
Dose-response relationship between dietary anthocyanidin intake and renal cancer risk, based on a restricted cubic spline, which adjusted for age, sex, race, body mass index, education level, smoking status, hypertension status, and total energy intake (*p* for nonlinearity = 0.207).

The restricted cubic spline model revealed a reduced risk of renal cancer with a higher anthocyanidin intake (Figure 1). There was no statistical evidence for nonlinearity (*p* for nonlinearity = 0.207). In the subgroup analyses as shown in Table 4, a significant interaction was observed between anthocyanidin consumption and hypertension status (*p* = 0.002). Specifically, the favorable association between anthocyanidin intake and renal cancer risk was more pronounced in the participants with a history of hypertension than in those without. No significant interaction was observed for other stratification factors including sex, BMI and smoking status (all *p* > 0.05). In a sensitivity analysis, there was little change in the findings by excluding individuals with a follow-up of less than two years (HR_Q4vsQ1_: 0.68; 95% CI: 0.51–0.92).

## 4. Discussion

In this post hoc analysis of the PLCO trial, we found that the dietary intake of total anthocyanidins was inversely associated with renal cancer risk, and this association was also true for the subclasses including delphinidin, peonidin and petunidin. Importantly, the association was evident in the dose-response analysis and the sensitivity analysis.

In the subgroup analyses, a significant interaction was observed between anthocyanidin consumption and hypertension status. A more favorable association between anthocyanidin intake and renal cancer risk was observed in participants with hypertension. The reason for these findings was not clear. Participants with a history of hypertension may be more likely to have a healthy lifestyle and dietary pattern, which can promote the intake of anthocyanidin-rich foods. No significant interaction was observed for other important renal cancer risk factors including high BMI and smoking behavior. In the subgroup analysis according to the subclasses of anthocyanidins, a significant association was only observed for half of the types of anthocyanidins, including delphinidin, peonidin, and petunidin, which indicated that only part of the subclasses of anthocyanidins have health benefits.

According to the literature reviewed, the evidence has accumulated worldwide on the beneficial effects of anthocyanidins on chronic diseases [19,20], including cardiovascular disease [21], diabetes [22], nonalcoholic fatty liver disease [23], and neurological disease [24], as well as various types of cancer. Zhang et al. [9] recently reported that dietary intake of total anthocyanidins and of all six subclasses including cyanidin, delphinidin, malvidin, peonidin, petunidin, and pelargonidin were related to a reduced risk of lung cancer in the PLCO cohort. A large meta-analysis of observational studies suggested an inverse association between anthocyanidin consumption and the risk of esophageal cancer [11]. Higher intake of dietary anthocyanidins may also reduce the risk of colorectal cancer [25]. However, the relationship between anthocyanidin intake and renal cancer risk remains unclear, especially given the lack of evidence from prospective studies.

Our study, firstly, provided important data on the association between dietary anthocyanidin intake and renal cancer risk from a large-scale prospective American cohort. We also examined the association visually in a dose-response manner and according to gender and subclasses of anthocyanins, with adjustment for potential confounders. Previously, there were only two small case-control studies performed on this topic with no significant association observed [12,26]. The reasons for the inconsistency in the findings between our study and the previous two are unknown. It could be due to the unknown residual confounding, insufficient statistical power, in addition to the selection bias and recall bias imposed by case-control studies.

Several mechanisms have been proposed to explain a potential inverse association between dietary anthocyanin intake and cancer incidence. Anthocyanins may influence the composition of the gut microbiome, which may mediate the metabolic benefits of anthocyanins [27]. In a mouse model, Khandelwal et al. [28] found that the intake of the anthocyanidins pelargonidin and cyanidin reduced the genotoxic stress induced by environmental toxicants, such as diepoxybutane, urethane and endogenous nitrozation. Li et al. [29] suggested that anthocyanins exhibited anticarcinogenic properties by suppressing the proinflammatory, STAT3, and NF-kB signaling pathways and promoting the activity of essential detoxification enzymes. Farhan et al. [30] proposed that anthocyanidins could suppress human cancers by trigger copper mediated and ROS-dependent selective cell death of cancer cells. A recent umbrella review summarized that anthocyanin improved plasmatic lipids, glucose metabolism, and endothelial function [31], which all may help in cancer prevention.

As with any study of this type, our study had several limitations. Firstly, anthocyanidin consumption was only assessed once at baseline in the PLCO study and dietary information may have changed over time. In addition, anthocyanidin consumption was assessed by a self-administrated FFQ in our study, which was typically prone to response bias. Secondly, although we had adjusted for a wide range of potential confounders, our results could be susceptible to residual confounding as the present study was performed with an observational design. Thirdly, the potential co-linearity between anthocyanidins and other nutrients could mediate the observed associations, which was not determined in this study. Finally, physical activity has been inversely associated with the renal cancer risk [32,33]. Long-term dialysis [34] was a potential risk factor for renal cancer. However, these data were not available in the PLCO study and thus we could not adjust for these potential confounders.

## 5. Conclusions

In conclusion, in this large American population, a higher dietary consumption of total anthocyanidins, as well as three subclasses including delphinidin, peonidin and petunidin, was associated with a lower risk of renal cancer. Future cohort studies are warranted to verify our preliminary findings and to explore the underlying mechanisms in this regard.

## Figures and Tables

**Table 1:** Main characteristics of participants included in this study by dietary anthocyanidin intakes.

Variables	Q1 (*n* = 25,340)	Q2 (*n* = 25,258)	Q3 (*n* = 25,269)	Q4 (*n* = 25,289)	*p*
Age (years), mean (SD)	61.9 (5.2)	62.5 (5.3)	62.6 (5.3)	62.6 (5.3)	<0.001
Sex (n, %)					<0.001
Male	14,499 (57.2%)	12,854 (50.9%)	11,712 (46.3%)	9996 (39.5%)	
Female	10,841 (42.8%)	12,404 (49.1%)	13,557 (53.7%)	15,293 (60.5%)	
Smoking (n, %)					<0.001
Never	10,072 (39.7%)	12,067 (47.8%)	12,868 (50.9%)	13,348 (52.8%)	
Current	4101 (16.2%)	2218 (8.8%)	1688 (6.7%)	1309 (5.2%)	
Former	11,166 (44.1%)	10,969 (43.4%)	10,709 (42.4%)	10,628 (42.0%)	
Education (n, %)					<0.001
≤High school	12,229 (48.3%)	10,844 (42.9%)	9944 (39.4%)	9597 (37.9%)	
≥Some college	13,064 (51.6%)	14,366 (56.9%)	15,273 (60.4%)	15,643 (61.9%)	
BMI (n, %)					<0.001
<25.0 kg/m^2^	7543 (29.8%)	8055 (31.9%)	8814 (34.9%)	9879 (39.1%)	
≥25.0 kg/m^2^	17,442 (68.8%)	16,875 (66.8%)	16,123 (63.8%)	15,093 (59.7%)	
Race (n, %)					<0.001
White, Non-Hispanic	22,592 (89.2%)	23,174 (91.7%)	23,305 (92.2%)	22,949 (90.7%)	
Other	2737 (10.8%)	2075 (8.2%)	1956 (7.7%)	2331 (9.2%)	
Hypertension (n, %)					<0.001
Yes	16,734 (66.4%)	16,839 (67.0%)	17,065 (67.9%)	17,188 (68.3%)	
No	8471 (33.6%)	8307 (33.0%)	8077 (32.1%)	7977 (31.7%)	
Total energy intake (kcal/d), mean ± SD	1510.0 (678.3)	1644.9 (663.5)	1767.4 (673.4)	1984.6 (712.0)	<0.001

y, year; SD, Standard deviation; BMI, body mass index.

**Table 2:** Association between dietary anthocyanidin intakes and renal cancer risk.

Variables (mg/day)	Median (mg/day)	Cohort (n)	Cases (n)	Crude HR (95% CI), *p*-Value	Adjusted HR * (95% CI), *p*-Value
Q1 (<6.95)	4.45	25,340	133	Reference	Reference
Q2 (≥ 6.95 to <12.18)	9.43	25,258	109	0.80 (0.62–1.03), *p* = 0.083	0.85 (0.66–1.10), *p* = 0.213
Q3 (≥ 12.18 to <20.21)	15.53	25,269	85	0.62 (0.47–0.81), *p* = 0.001	0.68 (0.51–0.90), *p* = 0.007
Q4 (≥20.21)	28.55	25,289	82	0.59 (0.45–0.78), *p* < 0.001	0.68 (0.51–0.92), *p* = 0.012
				*p* for trend < 0.001	*p* for trend = 0.010

* Adjusted for age (continuous), sex (male vs. female), race (non-Hispanic White vs. Other), body mass index (BMI, <25.0 kg/m^2^ vs. ≥25.0 kg/m^2^), education (≤high school vs. ≥some college), smoking status (never vs. former vs. current), hypertension (yes vs. no), and total energy intake (continuous).

**Table 3:** Associations between the subclasses of anthocyanidin intakes and renal cancer risk.

Variables	Cyanidin	Delphinidin	Malvidin	Pelargonidin	Peonidin	Petunidin
Q1	Reference	Reference	Reference	Reference	Reference	Reference
Q2	0.83 (0.63–1.10), *p* = 0.192	0.78 (0.59–1.03), *p* = 0.075	1.02 (0.78–1.34), *p* = 0.874	0.90 (0.69–1.18), *p* = 0.445	0.97 (0.74–1.28), *p* = 0.852	0.99 (0.76–1.29), *p* = 0.916
Q3	0.80 (0.60–1.07), *p* = 0.128	0.85 (0.65–1.11), *p* = 0.225	0.98 (0.75–1.30), *p* = 0.906	0.93 (0.70–1.23), *p* = 0.606	1.06 (0.81–1.38), *p* = 0.686	0.92 (0.70–1.22), *p* = 0.556
Q4	0.86 (0.65–1.15), *p* = 0.323	0.59 (0.43–0.79), *p* < 0.001	0.75 (0.55–1.02), *p* = 0.069	0.79 (0.58–1.06), *p* = 0.120	0.68 (0.50–0.93), *p* = 0.016	0.69 (0.50–0.94), *p* = 0.020

Adjusted for age (continuous), sex (male vs. female), race (non-Hispanic White vs. Other), body mass index (BMI, <25.0 kg/m^2^ vs. ≥25.0 kg/m^2^), education (≤high school vs. ≥some college), smoking status (never vs. former vs. current), hypertension (yes vs. no), and total energy intake (continuous).

**Table 4:** Subgroup analyses between dietary anthocyanidin intakes and renal cancer risk.

Variables	Q1	Q2	Q3	Q4	*p* for Interaction
Sex					> 0.05
Male	Reference	0.95 (0.70–1.29), *p* = 0.738	0.70 (0.49–0.99), *p* = 0.044	0.73 (0.50–1.06), *p* = 0.094	
Female	Reference	0.66 (0.41–1.06), *p* = 0.082	0.62 (0.39–1.00), *p* = 0.051	0.58 (0.35–0.95), *p* = 0.031	
Smoking					> 0.05
Never	Reference	0.95 (0.62–1.46), *p* = 0.810	0.80 (0.51–1.25), *p* = 0.328	0.70 (0.43–1.13), *p* = 0.147	
Current	Reference	0.38 (0.17–0.88), *p* = 0.023	0.22 (0.07–0.74), *p* = 0.014	0.38 (0.13–1.11), *p* = 0.077	
Former	Reference	0.97 (0.67–1.41), *p* = 0.886	0.75 (0.50–1.12), *p* = 0.159	0.82 (0.54–1.24), *p* = 0.339	
BMI (n, %)					> 0.05
<25.0 kg/m^2^	Reference	0.72 (0.41–1.28), *p* = 0.266	0.76 (0.43–1.35), *p* = 0.353	0.70 (0.38–1.29), *p* = 0.253	
≥25.0 kg/m^2^	Reference	0.89 (0.66–1.19), *p* = 0.420	0.66 (0.48–0.92), *p* = 0.013	0.69 (0.49–0.97), *p* = 0.032	
Hypertension					0.002
Yes	Reference	0.55 (0.38–0.81), *p* = 0.003	0.66 (0.46–0.96), *p* = 0.031	0.61 (0.41–0.92), *p* = 0.017	
No	Reference	1.27 (0.88–1.82), *p* = 0.196	0.70 (0.46–1.08), *p* = 0.111	0.79 (0.51–1.24), *p* = 0.306	

BMI, body mass index.

## Data Availability

The data used in this study can be applied from PLCO website (https://cdas.cancer.gov/datasets/plco/, accessed on 15 August 2022).
